# Interpretable Machine Learning Methods for Monitoring Polymer Degradation in Extrusion of Polylactic Acid

**DOI:** 10.3390/polym15173566

**Published:** 2023-08-28

**Authors:** Nimra Munir, Ross McMorrow, Konrad Mulrennan, Darren Whitaker, Seán McLoone, Minna Kellomäki, Elina Talvitie, Inari Lyyra, Marion McAfee

**Affiliations:** 1 Centre for Mathematical Modelling and Intelligent Systems for Health and Environment (MISHE), Atlantic Technological University, ATU Sligo, Ash Lane, F91 YW50 Sligo, Ireland; konrad.mulrennan@atu.ie; 2 Centre for Precision Engineering, Materials and Manufacturing (PEM Centre), Atlantic Technological University, ATU Sligo, Ash Lane, F91 YW50 Sligo, Ireland; 3 Department of Mechatronic Engineering, Atlantic Technological University, ATU Sligo, Ash Lane, F91 YW50 Sligo, Ireland; ross.mcmorrow@atu.ie; 4 Perceptive Engineering-An Applied Materials Company, Keckwick Lane, Daresbury WA4 4AB, UK; darren_whitaker@amat.com; 5 Centre for Intelligent Autonomous Manufacturing Systems, Queen’s University Belfast, Belfast BT7 1NN, UK; s.mcloone@qub.ac.uk; 6 Biomaterials and Tissue Engineering Group, Faculty of Medicine and Health Technology, BioMediTech, Tampere University, 33720 Tampere, Finland; minna.kellomaki@tuni.fi (M.K.); elina.t.talvitie@gmail.com (E.T.); inari.lyyra@tuni.fi (I.L.)

**Keywords:** feature selection, data summarisation, process monitoring, PLA, NIR, polymer degradation, extrusion, soft sensor, molecular weight, iPLS

## Abstract

This work investigates real-time monitoring of extrusion-induced degradation in different grades of PLA across a range of process conditions and machine set-ups. Data on machine settings together with in-process sensor data, including temperature, pressure, and near-infrared (NIR) spectra, are used as inputs to predict the molecular weight and mechanical properties of the product. Many soft sensor approaches based on complex spectral data are essentially ‘black-box’ in nature, which can limit industrial acceptability. Hence, the focus here is on identifying an optimal approach to developing interpretable models while achieving high predictive accuracy and robustness across different process settings. The performance of a Recursive Feature Elimination (RFE) approach was compared to more common dimension reduction and regression approaches including Partial Least Squares (PLS), iterative PLS (i-PLS), Principal Component Regression (PCR), ridge regression, Least Absolute Shrinkage and Selection Operator (LASSO), and Random Forest (RF). It is shown that for medical-grade PLA processed under moisture-controlled conditions, accurate prediction of molecular weight is possible over a wide range of process conditions and different machine settings (different nozzle types for downstream fibre spinning) with an RFE-RF algorithm. Similarly, for the prediction of yield stress, RFE-RF achieved excellent predictive performance, outperforming the other approaches in terms of simplicity, interpretability, and accuracy. The features selected by the RFE model provide important insights to the process. It was found that change in molecular weight was not an important factor affecting the mechanical properties of the PLA, which is primarily related to the pressure and temperature at the latter stages of the extrusion process. The temperature at the extruder exit was also the most important predictor of degradation of the polymer molecular weight, highlighting the importance of accurate melt temperature control in the process. RFE not only outperforms more established methods as a soft sensor method, but also has significant advantages in terms of computational efficiency, simplicity, and interpretability. RFE-based soft sensors are promising for better quality control in processing thermally sensitive polymers such as PLA, in particular demonstrating for the first time the ability to monitor molecular weight degradation during processing across various machine settings.

## 1. Introduction

In line with the general trend in manufacturing, polymer processing equipment is increasingly being sensorised for better real-time monitoring in expensive and complex material processing, such as in medical and pharmaceutical applications. In particular, spectroscopic instruments such as NIR, Raman, and UV-Vis, which are sensitive to molecular bond activity and/or physical morphology, have been shown to be valuable tools for real-time monitoring of variables such as additive concentration, degradation, mechanical properties, and particle size [1,2,3,4,5]. Such instruments together with increasing numbers of more conventional sensors such as pressure and temperature transducers result in high-dimensional data sets, which are used in conjunction with machine learning algorithms to generate predictive models from training data. Such approaches show great potential to improve product quality and reduce waste and downtime in polymer processing industries [6,7]. However, a drawback of many of the machine learning (ML) approaches advocated for in the literature is the black-box nature of such algorithms. In many cases, it is difficult to tell *why* the model is able to make accurate predictions, and they give little insight into the underlying process factors and relationships that influence the product quality. In highly regulated industries such as pharmaceutical and medical devices, this lack of model ‘interpretability’ is a barrier to industrial acceptance, as it is difficult to trust in the long-term robustness of a black-box model with little insight into what the predictions are based on [8]. Most of the research on ‘soft sensors’ for polymer processing utilises the linear regression methods of Principal Component Regression (PCR) and Partial Least Squares (PLS), while other works have explored more complex nonlinear models such as Artificial Neural Networks (ANNs), Random Forests (RFs), and Support Vector Machines (SVMs) [7]. However, these methods obscure the importance of individual variables and measurements and do not provide any insight into the process or how to correct deviations when they arise. Further, usually only small data sets are available at the process development stage, while many ML algorithms commonly applied for process monitoring and optimisation require large data sets for robust results [9]. Feature selection methods such as Recursive Feature Elimination (RFE), Forward Feature Selection (FFS), etc., which involve selecting only a subset of the original data features for use in a regression model, can yield simpler and more interpretable models but have not previously been explored for application in polymer manufacturing processes.

A challenging, yet valuable, class of materials in the polymer processing industry is that of thermally sensitive, biodegradable polyesters such as Polylactic acid (PLA). PLA has been widely used in pharmaceutical and medical products as well as in biodegradable packaging. PLA is used to make implantable medical devices for drug delivery, tissue scaffolds, stents, etc. [10,11]. Like other heat-sensitive polymers, PLA tends to degrade in the presence of high heat and mechanical stresses, which can cause unpredictability in the resulting mechanical properties and degradation rate. In the hot-melt extrusion (HME) process, the main process variables are feed rate, screw speed, and barrel temperature. The effect of these variables on the polymer product is complex and can vary from batch to batch and across different extrusion equipment. At high temperature and screw speed conditions, heat-sensitive polymers and polymer–drug compounds (for medical and pharmaceutical applications) tend to degrade [12,13]. However, at low screw speed, the residence time increases, which means that the polymer is exposed to heat and mechanical stress for a more extended period, which may also result in degradation [13]. In-process degradation affects the final properties of the extrudate including crystallinity, mechanical properties, drug purity, and dissolution properties [14]. Typically, these properties can only be determined via time-consuming laboratory tests. Novel tools for monitoring changes in key quality variables such as the polymer molecular weight or product mechanical properties in real time are highly desired. Despite the potential benefits of using in-line sensors coupled with machine learning algorithms for real-time process monitoring, black-box modelling approaches such as those previously described in the literature (RF, ANN, SVM, etc.) have had little uptake in practical industrial processes. This is mostly due to low confidence in model robustness associated with the lack of interpretability of these models. Feature Selection methods, on the other hand, may help to improve process understanding and control, as well as enhance acceptance levels for soft sensor approaches in industry. 

Mechanical properties are important for both medical and packaging applications of PLA. Degradation during the HME process reduces the molecular weight, which in turn can reduce the mechanical properties as well as influencing the rate at which the mechanical properties deteriorate over the product lifetime. Some studies have been reported in the literature focusing on prediction of mechanical properties and degradation of polymers from in-process data, typically with fixed processing conditions and machine set-up. Mulrennan et al. [15] used in-process NIR data along with pressure and temperature data to predict the yield stress of an extruded PLA product and achieved excellent accuracy on an independent test set using the nonlinear approaches of RF and SVM preceded by principal component dimension reduction. Wang et al. [16] used in-line UV-Vis for qualitative monitoring of the reduction in molar mass of Poly-L-Lactic Acid (PLLA). However, in this work, they did not apply any machine learning algorithm for the quantitative prediction of molecular weight. Montano-Herrera et al. [4] used in-line NIR coupled with PLS to monitor the degradation of four differently treated Polyhydroxyalkanoates (PHAs) and achieved good correlation with offline assessment of the degradation. Guo et al. [3] used in-line Raman to predict the degradation of polypropylene (PP) over repeated extrusion cycles conducted under the same processing conditions. The degree of degradation was calculated by comparing the molecular weight of PP following each extrusion run with the initial molecular weight (before extrusion). PLS showed good ability to predict the degree of degradation. 

Most of the works reported in the literature to date investigate the effect of feed material variations on degradation [6]. In such works, processing conditions are kept constant to avoid confounding the effect of chemical changes on the spectra with changes due to differences in temperature or other physical factors. This limits the usefulness of such approaches as new models must be created for any change in processing conditions. Further, the majority of previous works are based on PLS and PCA methods. These yield simple, low-dimensional linear models that perform well for many problems, but they can lack interpretability. For example, in the case of PLS, the latent variables in the resulting transformed data set are linear combinations of all of the original variables. In the final PLS model, many highly correlated and redundant latent variables are removed but it is difficult to infer the relationship between the predicted response and the original input features. This can obscure physical understanding of what factors are important in terms of achieving the required product quality [17,18]. 

Feature selection methods, in contrast, select a subset of the original features (e.g., specific spectral wavelengths or temperature at a particular point in the process) to use in a model. The most relevant features, i.e., features that give optimal predictions of the response variable of interest, are selected, and redundant and irrelevant features are eliminated from the data [19]. Feature selection methods help to reduce model complexity, reduce overfitting, and improve model predictive accuracy as well as the interpretability of the model. PCA achieves all the same things as feature selection methods and generally will be much quicker to compute than feature selection methods. However, as aforementioned, PCA does not provide information about key process variables and lacks interpretability, while feature selection methods identify key process variables that help to understand, control, and optimise the process. Feature selection methods have been used in bioinformatics [20] and in various applications in the medical industry as well, e.g., image processing, DNA microarray data analysis, and biomedical signal processing [21]. 

Investigation of the potential benefits of feature selection methods over latent variable approaches such as PCA has not previously been reported in monitoring of polymer processes. In this work, we explore the potential for feature selection methods to predict the mechanical properties and molecular weight of PLA grades from complex in-process data in real time. Good predictions of the mechanical properties of PLA from in-process data have previously been reported using a PCA-RF model [15]. However, here, we investigate optimal data summarization and dimension reduction approaches leading to significant improvement in performance. It is shown that combining several summary statistics with Recursive Feature Elimination (RFE) outperforms classical dimension reduction methods while resulting in a simple, sparse model giving insight to the key process variables and parameters. In some cases, selected features defy expectations based on prior process knowledge. Further, the real-time prediction of molecular weight in the processing of both packaging-grade and medical-grade PLA is investigated at varying temperature and screw speed settings, as well as with different machine set-ups (different nozzles for forming polymer fibres). This is a significant extension of prior works limited to fixed machine configuration and settings, which are therefore only useful once the optimum process set-up has already been identified [3,4]. Again, RFE is shown to outperform the more common latent variable methods and to achieve excellent prediction of product molecular weight across different machine set-ups and conditions in the case of medical-grade PLA. A key advantage is that the features selected by RFE give important insights into the primary parameters that should be controlled to prevent excessive degradation of mechanical properties and molecular weight during processing. 

The rest of this paper is organised as follows: Section 2 presents a background overview of regression and feature selection methods; Section 3 presents the methodology encompassing extrusion trials, model development, and data analysis; Section 4 presents the results and discussion of mechanical property predictions; Section 5 presents the results and discussion of molecular weight prediction for both PLA grades; Section 6 presents the conclusions.

## 2. Regression and Feature Selection Methods

This section provides a brief overview of the algorithms investigated in this work. 

### 2.1. Feature Selection Methods

Feature selection methods reduce the size of a data set by keeping features with relevant information and discarding the redundant features from the data set. Based on the feature selection criterion, feature selection methods are divided into different categories including wrapper, filter, and embedded approaches. Filter-type feature selection methods are used as a pre-processing step. Different types of statistical tests, e.g., ANOVA, chi-square, etc., are used to select the features based on their importance score with relevance to response variables. A model is then trained with selected features; thus, feature selection in filter-type methods is not related to model training but rather occurs as a preceding step. Wrapper methods train a model with a subset of features and then add/remove features from the model using a selection criterion. At each step, the performance of the model is measured after the addition/removal of the feature. Forward selection, backward elimination, and Recursive Feature Elimination (RFE) are wrapper methods. In embedded methods, feature selection is implemented as part of the model learning process, e.g., LASSO [19,20,21]. LASSO (Least Absolute Shrinkage and Selection Operator) is a regularisation method used to handle multicollinearity problems of linear regression [22]. Regularisation methods are a modification of the ordinary least square (OLS) method. LASSO imposes a penalty on the sum of the absolute magnitudes (L1-norm) of the regression coefficients [22], driving some of the coefficients to zero, thereby eliminating these features from the model. Further details of how each method works, advantages and disadvantages of different feature selection methods, and applications can be found in [19,20,21]. In this work, we employed RFE, which is a backward elimination approach. RFE has an advantage over Forward Feature Selection as it considers the effect of all variables simultaneously and so assesses the combined predictive ability of variables, unlike forward selection, which starts with a null/empty model [23]. However, as backward elimination starts with all variables, it can be computationally intensive compared to forward selection. The results of feature selection methods are compared with LASSO, which is a commonly used feature selection method in the literature. 

### 2.2. Recursive Feature Elimination

**Figure 1:**
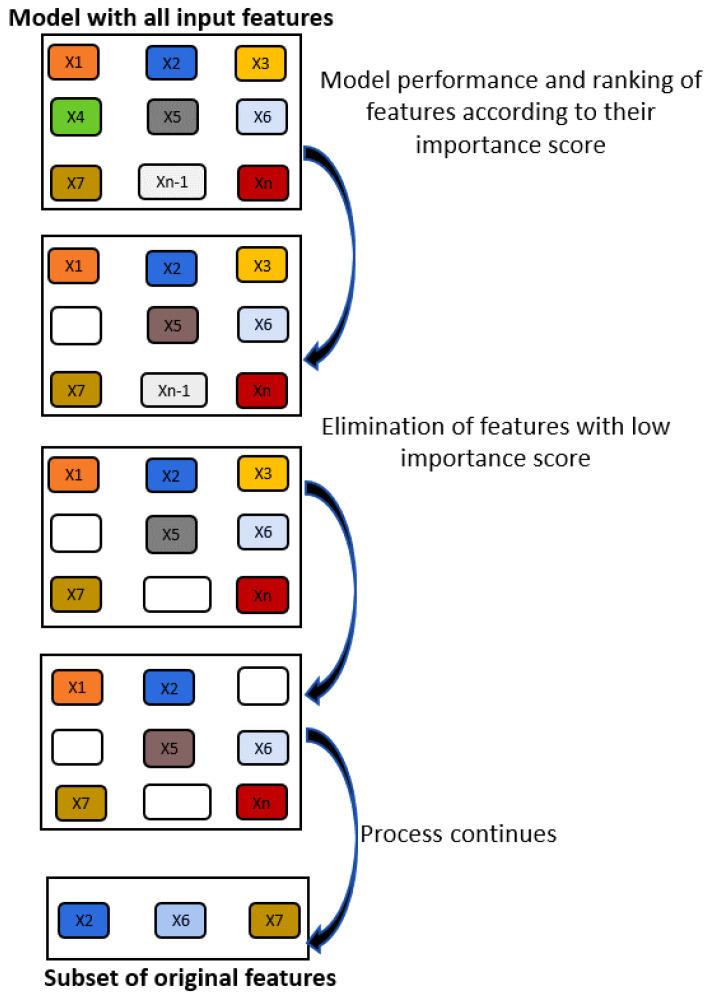
Schematic representation of RFE process.

Recursive Feature Elimination (RFE) is a backward feature elimination technique. RFE starts with all features, and a learning algorithm ranks the features according to their importance at each iteration. Features with low importance scores are discarded from the model at each stage of the search. The performance of the model is assessed with the retained features. This process continues until the model reaches a pre-defined number of features. Figure 1 shows the schematic representation of the RFE process. Different learning algorithms can be used with RFE, such as RF, bagging, SVR, linear regression, etc. It should be noted that both RFE and sequential backward feature selection are backward elimination methods, i.e., both start with all variables and then remove features from the model one by one. In RFE, features are ranked according to their importance score by the learning algorithm, while in sequential backward elimination, variables are selected based on their *p*-value. Variables with a *p*-value higher than the cut off *p*-value are discarded from the model. The model is refitted with the remaining variables and *p*-values are recomputed; this process continues until the model that remains only has significant features [22,24]. In this work, RFE was used with RF and bagging. In RFE, the size of the predictor subset for performance evaluation is pre-defined; thus, the size of the subset and the learning algorithm applied can be used as tuning parameters. 

### 2.3. Interval PLS (iPLS)

Interval PLS (iPLS) is a feature selection approach for spectroscopic data to select only relevant regions of the spectrum and ignore uninformative regions [25]. iPLS divides the whole spectrum into a given number of equally sized intervals and builds local PLS models for each interval. The performance of the local PLS models and a global PLS model (with full spectrum) is compared in terms of Root Mean Square Error of Cross-Validation (RMSECV). The main idea is to investigate the combination of different regions of spectral data to find the most relevant spectral regions and remove the irrelevant spectral regions. iPLS is further divided into two classes: forward and backward. Forward interval PLS (FiPLS) starts by splitting the spectra into a given number of intervals and then creating an empty vector. At each step, one spectral region is added to the vector, which is selected on the basis of the best performance of the resulting PLS model. This process continues until no further improvement is obtained [25]. Backward interval PLS (BiPLS) works on the same principle with the difference that initially, all the intervals are included, and instead of adding a new interval, it removes the poorest-performing interval at each step [26]. In this work, the number of intervals to divide the full spectrum was used as a tuning parameter and varied between 5 and 30 (with an increment of 5). In this work, iPLS approaches were applied to remove redundant regions of the spectra and were followed by RFE to select individual wavelengths from the remaining regions. 

### 2.4. Regression Methods

PLS regression is a dimension reduction method in which a linear transformation of the original features is performed. Newly formed features, which are called ‘latent variables’, are formed from the independent matrix *X* that best predict the dependent variable *Y* along with explaining the maximum covariance between *X* and *Y* [27,28]. Statistically, PLS regression transforms a set of correlated variables into a new set of uncorrelated variables. Partial Least Square Regression (PLS) is arguably the most widely applied regression method in polymer and pharmaceutical applications. Principal Component Regression (PCR) is closely associated with PLS. PCR also involves linear regression. PCR decomposes the independent matrix *X* by performing principal component analysis on it and uses the principal components to predict the dependent variable *Y* [28]. 

Ridge regression, like LASSO, is a regularisation method and is used to handle multicollinearity problems of linear regression [22]. In ridge regression, a penalty equal to the square of the magnitude of the coefficients (L2-norm) is introduced in the cost equation of linear regression. In ridge regression, the magnitudes of the coefficients for uninformative or redundant features are reduced towards (but do not reach) zero, hence all features are retained in the model. In contrast, in LASSO, some of the coefficients are driven to exactly zero. While this can result in some loss of information, it creates a simpler and more interpretable model. As such, LASSO can be considered both a feature selection and a regression algorithm (embedded feature selection).

Bagging (‘bootstrap aggregation’) utilises an ensemble of decision trees to perform regression. In the bagging approach, the data for each tree are generated by creating a bootstrapped data set from the original training set. That is, several random samples are taken from original data set with replacement, which means the observations in the original data set can be sampled many times. The bagging model makes a prediction by using the average of the predictions from all the trees in the model. Random Forest (RF) is an extension of bagging. However, in the RF model, only a subset of input features can be selected at random for each decision tree, i.e., the size of the subset of input features to be used by the decision tree can be tuned. A Random Forest model searches for the best predictive features and split values from within that subset, and like bagging, the prediction is made using the mean of all trees [29,30,31,32]. Further details on ensemble methods can be found in [32].

### 2.5. Data Summarisation

A common challenge in building data-based predictive models of product quality from process data is the mismatch in sampling rate between different sensors and the off-line characterisation data used for model training. Usually, there are hundreds of in-process data points corresponding to only one value of the target variable. One of the ways to match the sampling rate of in-line data with off-line characterisation data is to summarise the data set using different summary statistics. This can help to reduce the size of the data set without losing important information [33]. However, one of the challenges associated with the data summarisation approach is deciding which summary statistics should be used to capture sufficient information from the process. Shah et al. [33] used a statistical pattern analysis approach (SPA) for analysing in-process spectral data. In-process spectral data were divided into various segments, and eight different summary statistics were applied to capture the information from each segment. The SPA-based model showed better predictive accuracy and robustness than commonly used PLS and LASSO models. 

In this work, we investigated three different data downsampling summarisation approaches and used various statistical measures to capture information from the process runs. The purpose of using various summarisation approaches (data downsampling) was to investigate if they are able to achieve good predictive accuracy without being too computationally expensive and provide information about key process variables when used with feature selection methods. 

## 3. Extrusion Trials and Data Preparation

**Figure 2:**
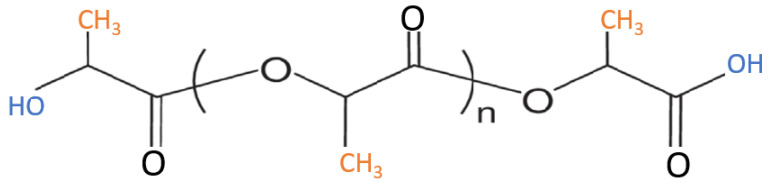
Chemical structure of PLA.

In this work, medical-grade and packaging-grade PLA were used. Both PLA grades have same polymer structure. The main difference is in the level of purity. Because of the nature of the end application, medical-grade PLA undergoes more rigorous purification processes to remove any type of impurity such as catalyst traces. Medical-grade PLA should meet strict regulatory standards set by the FDA to ensure its feasibility for medical applications [34]. While packaging-grade PLA is mostly used in food packaging and in biodegradable packaging for some other applications, it does not need to meet FDA standards. Figure 2 represents the chemical structure of PLA.

### 3.1. Packaging-Grade PLA Data Set

A data set relating to the extrusion of a packaging-grade PLA was used to investigate the prediction of the product molecular weight and yield stress and to explore whether feature selection algorithms could yield insight into the main factors governing both quality indicators. The experimental trials and yield stress results have previously been reported [15]. However, the effect of processing on degrading the molecular weight, and whether this can be inferred from in-process data, has not previously been investigated. Further, while good yield stress predictions have been achieved in prior work using PCA with the nonlinear regression algorithms of SVR and RF, these black-box models can struggle for industrial acceptance due to the lack of explainability, and a more interpretable modelling approach is preferred [35]. A brief overview of this data set is given here. Packaging-grade PLA (2003D, nature works, LLC) was extruded using a 16mm twin-screw extruder (TSE) under ambient environmental conditions. The temperature, screw speed, and feed rate were varied using a full factorial Design of Experiment methodology (DoE) with replicates. Two levels for feed rate (1160 g/h, 1600 g/h), two levels for screw speed (56 rpm, 83 rpm), and three levels for temperature (200, 210, 220 °C) were used. Twenty-four experiments were conducted initially, and six experiments, including with different temperature settings to those used in the original experiments, were conducted several months later and used as an independent test set. Three samples were taken from each process run to measure yield stress using a tensile testing machine. Molecular weight analysis has not previously been reported for this data set and we describe this process here. One sample from each process run was used to measure average molecular weight (Mw) using high-performance liquid chromatography (HPLC). Samples prepared in chloroform had a concentration of 2.5 mg/mL and were injected at a low rate of 0.8 mL/min. The mobile phase was chloroform. The columns used were an Agilent ResiPore Guard precolumn 50 × 7.5 mm and an Agilent ResiPore 300 × 7.5 mm. The column was kept at a temperature of 30 °C. The Dionex Ultimate 3000 HPLC system (Thermo-Scientific, Dreieich, Germany) and the detector (Varian 385-LC) was an evaporative light-scattering detector. The detector had an evaporator temperature of 50 °C, a nebuliser temperature of 50 °C, and a carrier low rate of 1.4 slm (standard litre per minute). The light source intensity was 10%. The system was calibrated using polystyrene standards 2000, 10,000, 30,000, 70,000, 150,000, and 300,000 Daltons. The injection volume of the samples was 10 µL.

#### Data Preparation for Packaging-Grade PLA to Predict Yield Stress and Molecular Weight

NIR data were collected for wavenumbers 4000–7500 cm^−1^ at a resolution of 4 cm^−1^. From the NIR data, only wavenumber ranges from 6100 to 6700 cm^−1^ (601 wavenumbers) were included as input features in the model, due to high noise levels in other regions of the spectra. The final model included pressure and temperature readings along with pressure drop across the adapter and a slit die, and shear viscosity estimates calculated from pressure drop across the die [36]. In total, 612 input features (including pressure and temperature data) were used as input features for packaging-grade PLA to predict yield stress and molecular weight. The in-line pressure and temperature data sample rate varied between 5 and 10 Hz. However, the NIR data capture period varied between 14 and 48 s. This resulted in more temperature and pressure data points than NIR measurements for each process run. A total of 50,762 data points for pressure and temperature, 371 NIR spectra, and 30 values of mean yield stress and Mw were collected. 

Three different summarisation approaches were compared to relate the in-process data to target variables. Table 1 lists the details of the summary methods for packaging-grade PLA used to predict yield stress and molecular weight, summary statistics used for each method, and size of the data set. In our previous work [37], we investigated sampling of data from short periods, i.e., at the start, middle, and end of a process run, but this approach did not achieve good results. We also investigated upsampling of all data points to the higher frequency using zero-order hold, but the application of RFE to this large data set was too computationally intensive to be practical. 

### 3.2. Medical-Grade PLA Extrusion Trials and Data Set

**Figure 3:**
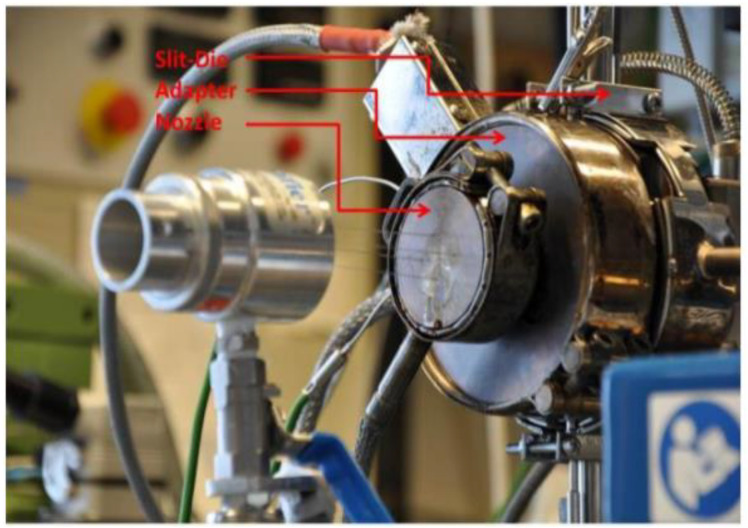
Extruder exiting zones for medical-grade PLA.

For medical-grade PLA, trials were conducted in the development of a fibre spinning process for a commercial tissue scaffold product. In-process data and process settings data were used to predict the molecular weight of an extruded medical-grade PLA product, as this is a crucial factor affecting the in vivo bioresorption rate for implantable devices such as sutures, bone screws and pins, and tissue scaffolds [38]. Medical-grade PLA is highly purified relative to packaging grades to avoid unwanted traces of catalysts and adverse degradation products. These extrusion trials were conducted using medical-grade PLA (PURAC biochem bv, Goringchem, Netherlands) using a co-rotating twin-screw extruder (Mini ZE 20 × 11.5 D, Neste Oy, Porvoo, Finland). To avoid oxidative degradation of PLA, these experiments were conducted in a nitrogen atmosphere. To avoid any moisture content in the PLA feedstock, the PLA was placed in a bottle and heated in a vacuum oven for 16 h, followed by cooling to room temperature. Then, the vacuum was replaced by a nitrogen atmosphere, and the bottles were sealed until the material was required for experiments. The extrusion system had four variable temperature zones: 1. barrel; 2. die; 3. adapter; and 4. nozzle (see Figure 3). Experiments were conducted using six different temperature profiles with a wide range of different feed rates. During all experiments, four different nozzle types were used, termed A, B, C, and D here. The nozzles differed from each other in terms of the number and size of holes. Moreover, for the analysis of molecular weight, for some experiments, samples were collected directly after extrusion, and in other cases, after downstream spooling of the fibres. All samples were characterised off-line to determine Mw using off-line gel permeation chromatography (GPC). The measurements were performed with a modular system (Shimadzu Corporation, Kyoto, Japan). Chloroform was used as an eluent at a flow rate of 1 mL/min. Samples were measured at 40 °C. The injection volume of the samples was 100 µL. The concentration of the samples was 0.1 mass %.

Table 2 provides a summary of the experimental details for these trials. Further details and the full data set and metadata are available for download [39]. The same slit die and sensors used in the packaging-grade trials were also used for in-process data capture for medical-grade PLA. 

#### Data Preparation for Medical-Grade PLA to Predict Molecular Weight

**Figure 4:**
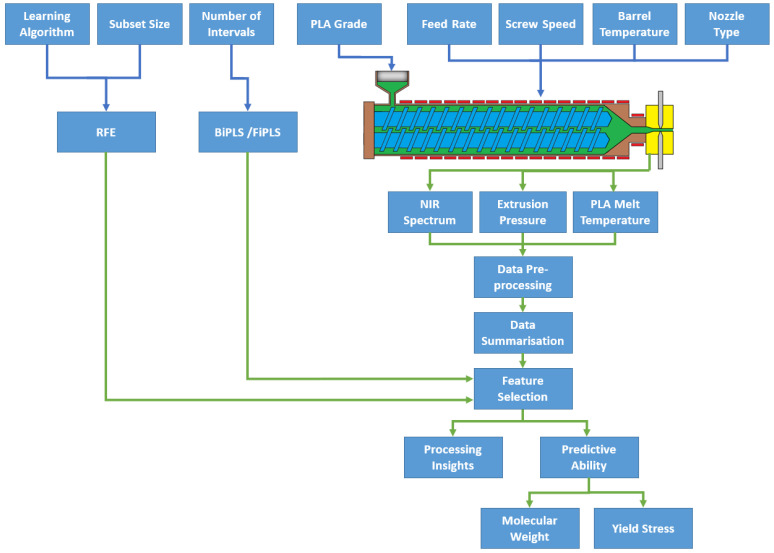
Schematic representation of the feature selection process.

For the prediction of molecular weight, models were developed using in-process data, including NIR, pressure, and temperature data. NIR data were included from 6100–6600 cm^−1^. Besides in-process data, process settings including the temperature of extruder barrel zones, throughput, feed rate, and nozzle sizes were included as input features in the model. For this data set, the number of samples (n) = 67 and number of input features (*p*) = 512. For medical-grade PLA, it was not possible to use summary methods with more statistics, e.g., variance, skewness, kurtosis, etc., as for some process runs, there were fewer than three NIR spectra recorded. For the sake of comparison, only summary method 3 was used for molecular weight prediction for the medical-grade and packaging-grade PLA data set. Table 1 defines the summary method and size of the data set used to predict molecular weight for medical-grade PLA. Figure 4 presents the schematic representation of this work. RStudio (version 2022.02.03+492) was used for model development and for data analysis.

## 4. Results and Discussion

### 4.1. Effect of Processing Conditions and Need for In-Process Monitoring

**Figure 5:**
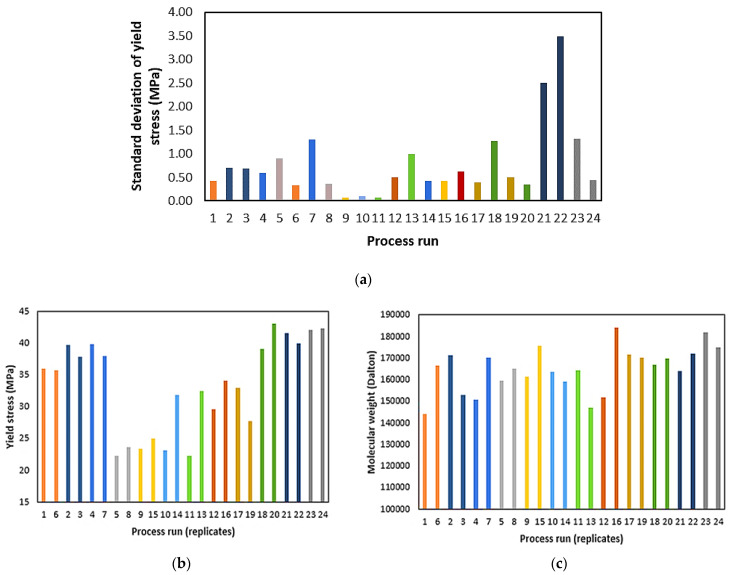
(**a**) Standard deviation between triplicate packaging-grade PLA for yield stress, (**b**) variation of mean yield stress, (**c**) variation of molecular weight between replicated process runs for packaging-grade PLA (process runs with identical settings are shown in the same colour).

**Figure 6:**
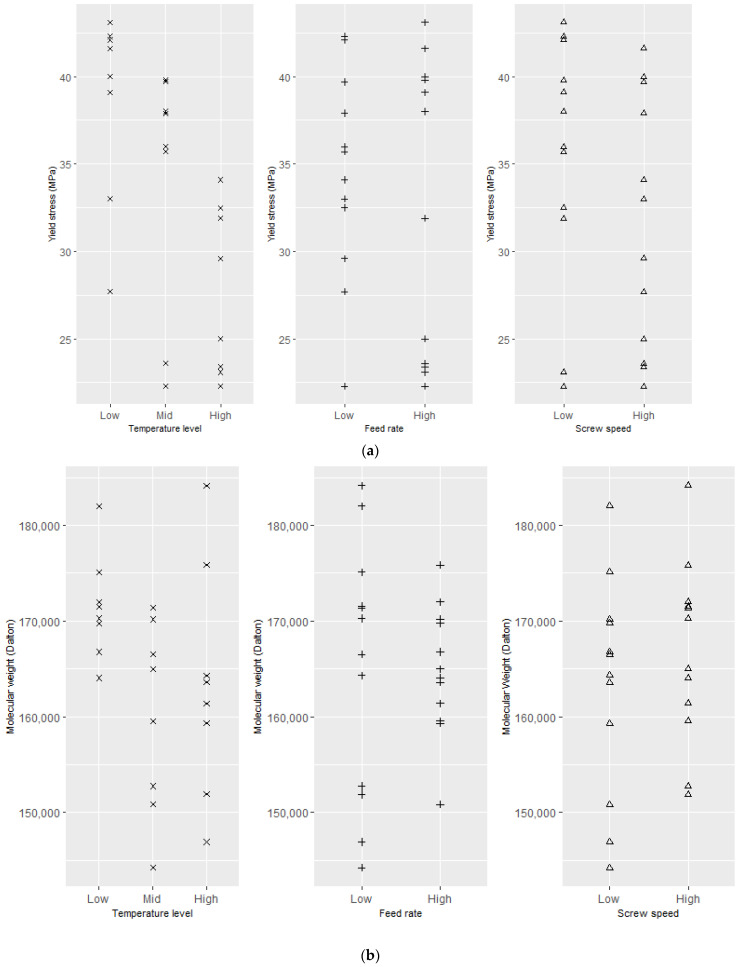
Effect of process settings on (**a**) yield stress (MPa) and (**b**) molecular weight for packaging-grade PLA (Dalton).

As described in the experimental section, for the packaging-grade PLA, each process run was replicated twice and three samples from each process run were used to measure yield stress. However, the effect of processing conditions on the resulting yield stress and molecular weight of the PLA was not consistent (see Figure 5b,c). Significant variations between some of the replicates (10,14; 11,13; 12,16; and 17,19) were observed. The standard deviation between triplicates for yield stress varied between 0.06 and 3.50 MPa for processes 1–24 (Figure 5a). Figure 6 shows the correlation between individual process variables and yield stress. No clear effect of screw speed or feed rate can be observed in these single-factor plots. In the case of temperature, a trend of decreasing yield stress with increasing temperature was observed. However, the correlation between temperature and yield stress is quite weak and some yield stress values deviated significantly from this trend. In the case of molecular weight for packaging-grade PLA, the effect of process variables on molecular weight was not consistent (see Figure 6b), and similar to the case of yield stress, variation between some of the replicated process runs was high (see Figure 5b,c). Overall, the effect of processing conditions on the yield stress was more pronounced than that on the molecular weight. 

**Figure 7:**
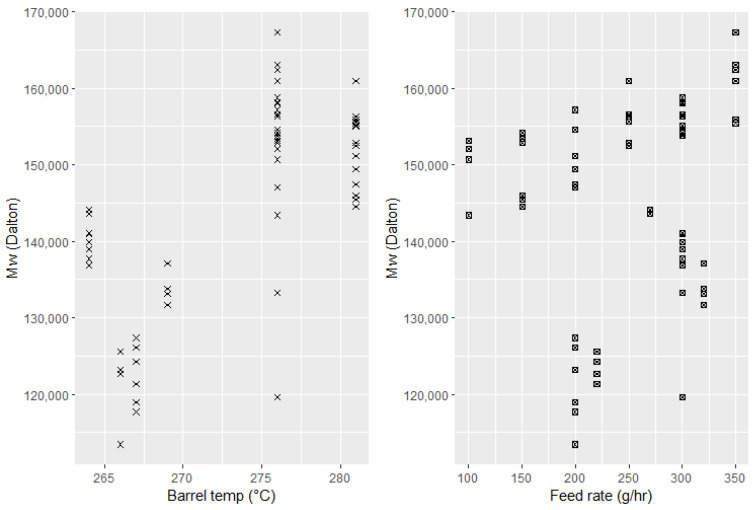
Effect of process settings on molecular weight of medical-grade PLA.

For medical-grade PLA, again, there is no strong correlation between the effect of individual process settings and molecular weight of the product. Here, there is a tendency for higher extrusion temperatures to result in higher Mw (see Figure 7). 

The reason for the inconsistent effect of processing conditions on the yield stress and molecular weight of PLA is attributed to the fact that PLA feedstock is very sensitive to environmental conditions (humidity), which can affect the final properties. In the case of HME, the effects of process conditions on the quality are complex and coupled, and hence it is not easy to determine from simple correlation analyses what corrective action should be taken to improve the product quality if it is found to be inadequate. It has been reported in the literature that mechanical stresses applied by the screws during the HME process generate heat inside the extruder, which can result in an actual melt temperature much higher than the set temperature [40,41]. It can be seen from these results that process settings are not enough to provide information about the final properties of the PLA product. In such cases, in-process measurements are important to provide real-time monitoring of the process.

### 4.2. Comparison of Data Summarisation and Machine Learning Methods to Predict Yield Stress

To compare the effect of the different summarisation methods, the performance of commonly used regression algorithms PLS, PCR, RF, and ridge regression were compared with feature selection methods including RFE-RF, RFE-bagging, and LASSO. These methods were used to predict yield stress for packaging-grade PLA for all summarised data sets. All algorithms were trained using process runs 1–24 conducted during initial experiments, and the lowest RMSE of 10-fold cross-validation (CV) was used for optimal hyperparameter selection for all models. For PLS and PCR, the number of components (latent variables, represented by N_c in Table 3) was increased in increments of one until it was clear that RMSECV passed a minimum point and no further reduction in RMSE was achieved. For LASSO and ridge, the regularisation tuning parameter λ (lambda) was increased from 0 to 3 in increments of 0.01. Random Forest was used with default features (caret package from RStudio, ntree = 500, mtry = *p* (no. of features)/3) without hyperparameter tunning. The predictive accuracy of all regression models was then investigated on the independent test set of six process runs conducted on a different day several months later. The tuning parameters for RFE were the learning algorithms and the size of the subset. The size of the subset is the maximum to the minimum number of features the model should evaluate at each iteration. A range of sizes of subsets was evaluated for RFE with RF and RFE with bagging. Table 3 lists the RMSE values of all the models for the independent test set. It can be seen from Table 3 that RFE-RF for summary method 1 (six stats) outperformed all other models and achieved the lowest RMSE value among all three summary methods with the least numbers of features. In Table 3, N_s represents the optimal number of features selected by the feature selection methods. 

RFE-bagging also achieved good predictive accuracy for all summary methods. For summary methods 2 and 3, RFE-bagging performed slightly better than RFE-RF. However, it also selected more features than RFE-RF for these summary methods. Furthermore, the bagging model is comparatively more complex than RF, and took 2.5 times longer to train. It can also be seen from Table 3 that none of the classic regression methods (PLS, PCR, RF, ridge) were able to achieve satisfactory accuracy for any of the data summarisation approaches. All summary methods yielded lower RMSE values when coupled with feature selection methods as compared to when applied without feature selection. 

The best performance was achieved with RFE using summary method 1 (with six summary stats). For the sake of comparison, this RFE result for yield stress prediction was compared with sequential Forward Feature Selection (FFS). FFS is expected to be more computationally efficient than backward elimination methods. FFS achieved reasonable accuracy but yielded a higher RMSE value than RFE with a higher number of features. For this data set, FFS and RFE required similar amounts of time to select the optimal set of features, so overall, RFE performed better than FFS.

As described in Section 2.2, iPLS is an approach to select relevant spectrum regions and remove redundant regions. In this work, we investigated whether a preceding iPLS step could help achieve better performance by removing redundant areas of the spectrum prior to RFE. FiPLS and BiPLS followed by RFE using RF and bagging were applied for summary method 1 as these approaches outperformed all others reported in Table 3. In the first step, iPLS was used to select the relevant regions of the spectrum. In the second step, the reduced set of wavelengths, together with the process data (temperature and pressure data), was included in the final model and RFE was applied using RF and bagging as learning algorithms. This method is termed FiPLS/BiPLS-RFE. For iPLS, the number of intervals to divide the whole spectrum varied between 5 and 30 (with increments of 5). For both BiPLS/FiPLS-RFE methods, 10-fold CV was used to select the optimal variables, and the accuracy of the selected variables was investigated using the independent test set. For both FiPLS and BiPLS, the best results were achieved using 20 intervals. Table 4 lists the RMSE values for the independent test set for FiPLS/BiPLS followed by RFE for method 1 (six summary stats). All methods performed quite well; however, the best results were achieved with BiPLS-RFE-bagging. BiPLS-RFE with bagging achieved a better RMSE value of 0.911 MPa than RFE-RF (1.07 MPa) without an iPLS step. However, it also selected eight more features than RFE-RF to achieve this RMSE value. The iPLS step improved the performance of the RFE-bagging model but not the RFE-RF model. FiPLS and BiPLS coupled with RFE were applied for all summarised data sets. However, only summary method 1 achieved a reasonable RMSE value; all other summary methods selected more features and yielded poor accuracy and hence the results are not presented here.

### 4.3. Interpretation of Selected Features

**Figure 8:**
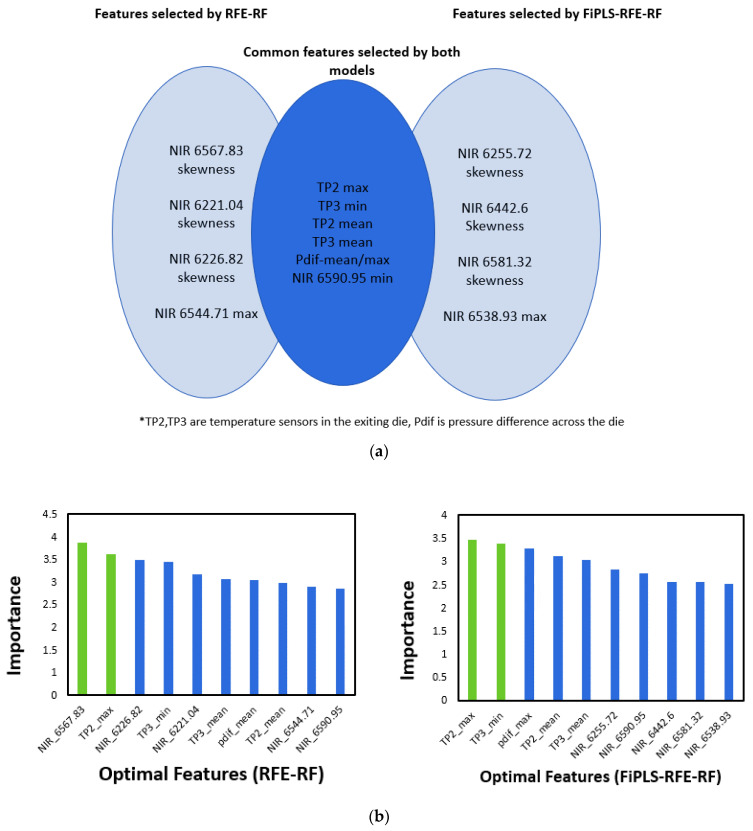
(**a**) Features selected by RFE-RF and FiPLS-RFE-RF, (**b**) importance scores of optimal features for the prediction of yield stress for packaging-grade PLA (TP2 and TP3 are melt temperatures at the entrance and exit to the die and pdif is pressure drop across the die between pressure transducers P2 and P3).

Besides achieving a sparse model with good predictive ability, another advantage of feature selection methods is that they provide important insights into the process in terms of providing information about key process variables. All the models reported in Table 4 selected almost similar optimal features: the temperature at the exit of the extruder die and the pressure drop across the die for prediction of the product yield stress. The die pressure drop is related to viscosity and shear rate changes. Figure 8a represents the features selected by two models (RFE-RF and FiPLS-RFE-RF), as these models achieved excellent predictive accuracy with the lowest number of features. Figure 8b presents the importance score of selected features. The features selected by RFE-RF and FiPLS-RFE-RF highlighted melt temperatures as the most critical variables. For semi crystalline polymers such as PLA, heating and cooling profiles affect the crystallinity of the polymer. Crystallinity has a major influence on the mechanical properties of the polymer products [42]. The selected features highlight the importance of temperature control at the end of the process to achieve the desired mechanical properties. Wavenumbers in the range of 6200 cm^−1^ to 6500 cm^−1^ were selected by both models. Interestingly, other statistical features (min, max, skewness) were found to be better predictors than the commonly used mean and variance. It can be difficult to relate NIR wavenumbers to specific molecular bond activities, due to the NIR range being dominated by combination and overtone effects [43]. However, the NIR features selected here are related to C-H bending (6221.04 cm^−1^, 6255.72 cm^−1^, 6422.5 cm^−1^, 6581.33 cm^−1^, 6538.93 cm^−1^, 6590.95 cm^−1^), C-H stretching of mode of CH_2_ (6567.83 cm^−1^), and C-H stretching of CH_3_ group (6226.82 cm^−1^). The intensity of C-H stretching of PLA is linked with the degree of crystallinity, molecular structure, and orientation of chains [44,45]; refer to Figure 2 to see the chemical structure of the PLA. These properties directly influence the mechanical properties of polymers [42]. It is worth highlighting that melt temperature varies throughout the experiments and the selection of particular wavelengths may also be due to the temperature sensitivity of particular bond activities. Changes in amplitude at these wavelengths may help to capture more detailed temperature changes in the melt, which are not captured by wall-mounted thermocouples [46].

## 5. Comparison of Data Summarisation and Feature Selection Methods to Predict Molecular Weight

### 5.1. Molecular Weight Prediction for Packaging-Grade PLA

The summarised data sets defined in Table 1 were used to predict the molecular weight of the PLA product (packaging grade). Experiments conducted on day 1 (24 experiments) were used to train the model, and 6 experiments conducted on a different day were used as an independent test set. Firstly, different algorithms including PLS, PCR, LASSO, and ridge regression were used for both summarised data sets to predict the Mw. As molecular weight units in Dalton results in high RMSE values, to present the results in a more readable form, an NRMSE (Normalised Root Mean Squared Error) and R^2^ values were used to evaluate the performance of the machine learning models. NRMSE was calculated by dividing the RMSE by the range of actual molecular weight values in the test set. R^2^ is the correlation coefficient between the actual and predicted values. None of the machine learning algorithms performed well and all methods yielded an extremely high NRMSE and extremely low R^2^ value for the independent test set.

### 5.2. Molecular Weight Prediction for Medical-Grade PLA

For the medical-grade PLA data set, no independent test was available, and an internal validation set was used to investigate the predictive accuracy of the machine learning models. As described in the data preparation section, some of the process runs for medical-grade PLA had fewer than three NIR spectra, so summary method 1 with many statistical measures (including kurtosis and skewness) and summary method 2 (mean and variance) were not appropriate. For medical-grade PLA, the total number of samples (n) was 62, and the number of input features (*p*) was 512 for summary method 3. In total, 80% of the data was used to train the model, and 10-fold CV was used to select the optimal features/model hyperparameters. The predictive accuracy of the selected features/parameters was investigated on the 20% unseen data. For Recursive Feature Elimination, the same hyperparameters explained in Section 2.2 were investigated. 

**Figure 9:**
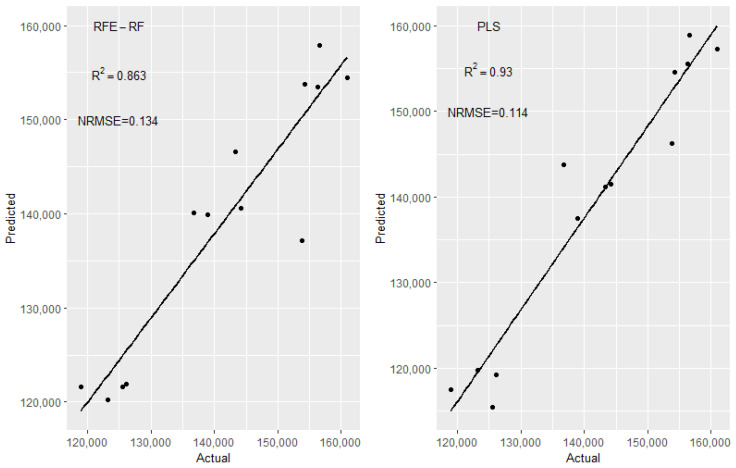
Molecular weight prediction in Daltons on unseen data for medical-grade PLA.

For molecular weight prediction for medical-grade PLA, among the feature selection methods, RFE-RF performed better than RFE-bagging and LASSO, as it achieved good predictive accuracy with fewer features. Other machine learning algorithms, including PLS, PCR, and ridge, were also applied. Among all methods, PLS and PCR performed slightly better than RFE-RF. However, with the RFE-RF approach, it is possible to obtain information about key process variables, unlike PLS and PCR. The results for all these methods are presented in Table 5. Figure 9 shows the linearity plots for the RFE-RF and PLS models, the two best-performing models for molecular weight predictions. 

In the absence of an independent test set, a more robust validation method is required. Instead of simply using a single 80:20 train test split, a more robust approach is to use Monte Carlo Cross-Validation (MC-CV). In MC-CV, a higher number of train–test iterations (here 200) can be used to eliminate the sensitivity of the model to a specific split in training and test data. At each iteration, 20% of the data is randomly selected as the test set to evaluate the predictive accuracy of the model trained on the other 80%. This gives 200 different values of R^2^ and RMSE, and the standard deviation of these values gives a measure of the robustness of the model. Mean NRMSE, standard deviation of NRMSE, and R^2^ value were measured. MC-CV was used for the best-performing models of RFE-RF, PLS, and PCR. As can be seen in Table 5, the mean NRMSE and R^2^ values resulting from MC-CV can be higher or lower than those found using a single split above, showing that a single split of training and test data may lead to under- or overestimating the more general performance of the model. The results show that RFE-RF has a lower mean NRMSE and a higher mean R^2^ than PLS and PCR, although it has a slightly higher standard deviation in NRMSE (c.17% vs. c.14%). It should also be noted that one of the limitations in molecular weight in the laboratory is that it is not a precise measurement even if measured by using GPC, as molecular weight has a distribution. Therefore, there will always be some variation in results, even for identical/the same samples.

**Figure 10:**
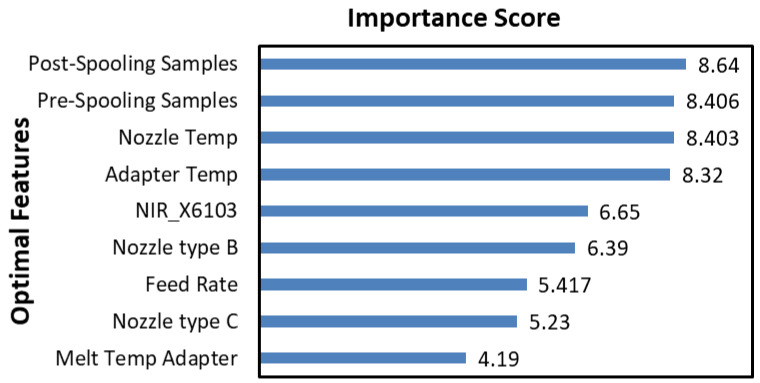
Importance score of optimal features for the prediction of molecular weight for medical-grade PLA.

Figure 10 represents the optimal features selected by RFE-RF and their importance score. The point at which samples were picked for molecular weight analysis (pre- or post spooling) has the most significant influence. This indicates that the downstream fibre spooling process likely causes some degree of degradation post extrusion. Of the machine settings, the optimal features selected included extruder adapter and nozzle temperatures, the type of nozzle, and extruder feed rate. From the NIR spectra, a wavenumber of 6103 cm^−1^ was selected. The melt temperature at the adapter was also highlighted as an influential parameter for the prediction of molecular weight. The selection of process temperatures from the last two zones of the extruder and the melt temperature at the adapter as optimal features again highlights the importance of controlling temperature at the end of the extruder section, as temperature affects the degradation rate, causing a reduction in the molecular weight of the PLA product. 

### 5.3. Comparison of Molecular Weight Prediction for Medical-Grade and Packaging-Grade PLA

As described in Section 5.1, none of the machine learning algorithms could achieve good predictive accuracy for the independent test set for packaging-grade PLA to predict molecular weight. As the molecular weight predictive ability was evaluated on an internal unseen data set for the medical-grade PLA, a similar evaluation process was also conducted for the packaging-grade data set for the sake of comparison. Here, the data from all thirty packaging-grade process runs were split in the same way as the medical-grade data set: 80% used to train the model and 20% used to investigate the predictive accuracy. However, all machine learning algorithms still resulted in a high NRMSE for the packaging-grade data set for the prediction of molecular weight. Table 5 compares the results for the 20% unseen data for both grades of material. For the packaging-grade PLA, process settings including set temperature for barrel zones, feed rate, and screw speed values were also included in the model as they were included for the medical-grade model, but this had no significant impact on the predictive performance. From the results, it can be seen that the machine learning models were able to predict molecular weight accurately over a wide range of conditions for medical-grade PLA but showed poor accuracy for packaging-grade PLA. 

Prediction of molecular weight is important in the case of PLA because PLA tends to degrade during thermal processing in the presence of mechanical stresses and under hydrolytic conditions, etc. For medical devices, this can have a significant impact on the in vivo performance. During hot-melt extrusion, three main types of degradation can occur: thermal degradation, chemical degradation, and mechanical degradation. Thermal degradation occurs at high temperatures. It can further be divided into depolymerisation, random chain scission, and unzipping of substituent groups. Mechanical degradation occurs due to high mechanical stress, which can cause molecular chain scission. High mechanical stresses can also cause overheating and initiate thermal degradation. Chemical degradation is initiated by the presence of chemicals such as acids, bases, and solvents, gasses, excipients’ incompatibility, and moisture content in the extrusion process. Temperature is regarded as the most crucial process parameter that affects the degradation; other process parameters that can initiate degradation include screw speed and residence time [41]. 

**Figure 11:**
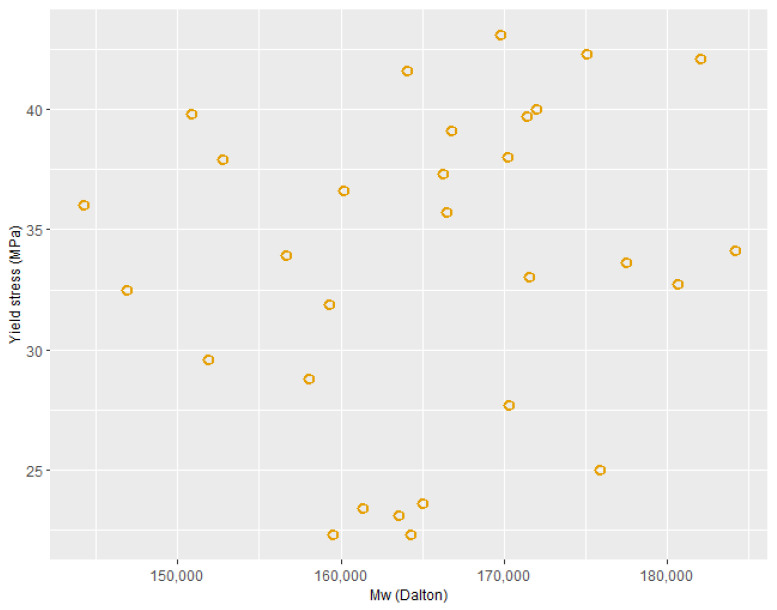
Molecular weight vs. yield stress for packaging-grade PLA.

PLA degradation affects the molecular weight, i.e., a rapid reduction in molecular weight occurs due to degradation, which affects the final properties, such as mechanical properties, of the PLA product [47,48,49]. It was anticipated that as the in-process data yielded high accuracy to predict the mechanical properties, it would also be possible to achieve good prediction for molecular weight data. However, as mentioned above, none of the algorithms could achieve reasonable accuracy to predict the molecular weight of the packaging-grade material. Mechanical properties of polymers such as modulus, stiffness, tensile strength, etc., are highly affected by the crystallinity of the polymers [42]. From this, it could be inferred that mainly heating during the process affected the crystallinity of the PLA, which in turn affected the yield stress. The degradation of molecular weight over the experimental processing window investigated here appeared not to significantly affect the mechanical properties of the PLA product. Figure 11 supports this hypothesis, as no clear relationship between molecular weight and yield stress could be observed. Unlike the case for yield stress, there was not a great deal of variation between the observed molecular weights for different process runs (see Figure 5b,c). This suggests that the processing window investigated for the packaging-grade material was appropriate in terms of avoiding excessive degradation of the material. 

The medical-grade data set used for the prediction of molecular weight was more complicated than the packaging-grade data set, as the medical-grade data set included data from experiments conducted over a wide range of processing conditions and machine settings. However, it was possible to predict the molecular weight of medical-grade PLA from the in-process data and process settings with good accuracy. The good performance achieved for the medical-grade data could be because the size of the data set for medical grade (n = 62) was twice the size of the packaging-grade data set (n = 30) and because a wider processing window was investigated for the medical-grade PLA. Experiments for medical-grade PLA were also conducted in more controlled conditions, minimising the risk of hydrolytic degradation compared to thermal/mechanical degradation. All medical-grade experiments were conducted in a nitrogen atmosphere while experiments for packaging-grade PLA were conducted in ambient conditions. Medical-grade PLA was also dried for longer than the packaging-grade material. A study using UV-vis spectroscopy to monitor PLLA degradation in extrusion processing found significant differences in results with dry and moist PLLA. Wang et al. [16] found a clear correlation between UV-vis absorption and molecular weight reduction for dry PLLA but not for PLLA containing moisture. Moisture content generally accelerates the degradation rate via hydrolytic degradation, and this was confirmed through off-line GPC. However, no significant effect could be observed on the UV-vis spectra. Hence, the mechanism of degradation has a significant effect on how well spectroscopic methods can detect the chemical changes. 

Overall, in this work, the Recursive Feature Elimination method achieved better predictive accuracy than classical dimension reduction and regression methods in predicting the yield stress for packaging-grade PLA and molecular weight for medical-grade PLA. RFE also provides important insight into the process by highlighting key process variables that influence the final properties of the PLA. 

It should be noted that feature selection approaches are generally very sensitive to how the data are split for training and testing, so a robust validation approach is required. An independent test set (as used for the packaging-grade PLA) is generally required for industrial validation. As no independent test set was available for the medical-grade data set, using the MC-CV approach with 200 resamples of training and validation data allows for analysing the robustness of the models to different splits in training and testing data. However, for industrial use, further independent trials would be required for validation. A drawback of summarizing the data set using extensive statistical features is that it is a time-consuming process. In future, the potential to use the models developed in this study for industrial process monitoring should be explored—in the first instance, as a tool to identify and correct process issues. With further data collection, it may be possible to use the real-time predictions to reduce the amount of off-line testing for quality control purposes. This holds the most potential for high-value medical and pharmaceutical products where quality control issues are of major concern and currently contribute to long lead times and high costs.

## 6. Conclusions

In this work, feature selection methods including Recursive Feature Elimination (RFE) with Random Forest, and bagging, and interval PLS (iPLS) were applied on three differently summarised data sets to predict yield stress and molecular weight in PLA processing. The RFE methods outperformed classical regression methods in accuracy metrics and give a simple model that helps to provide insight into the process. RFE performed best with rich statistical summarisation information, compared to summarising data with only mean and variance measures. A preceding iPLS step removed redundant regions of the spectrum but did not significantly improve performance over RFE-RF alone, which is more time-efficient. 

For the prediction of yield stress of packaging-grade PLA, RFE-RF achieved an RMSE value of 1.07 MPa and R^2^ of 91.2% on an independent test set. Ten features were selected by the algorithm and temperature at the end of the process was highlighted as the most important variable to affect the yield stress of the PLA product. From NIR data, wavelengths related to the C-H stretching and bending were highlighted as important variables. The intensity of C-H stretching/bending of PLA is linked with the degree of crystallinity, molecular structure, and orientation of chains (see Figure 2, PLA structure). These properties affect the mechanical properties of polymers. 

In the case of molecular weight prediction for packaging-grade PLA, none of the machine learning methods could achieve good predictive accuracy, highlighting that changes in molecular weight during the process are not related to the yield stress (as reported in Figure 11) and that changes in crystallinity of the PLA, which are not clearly observed in the process data, dominate the mechanical properties in this case. 

Conversely, for the prediction of molecular weight for medical-grade PLA, good accuracy was achieved from in-process and process setting data. RFE-RF performed better than the other methods, achieving a mean Normalised Root Mean Squared Error (NRMSE) of 0.101 (10.1%) and R^2^ of 83% with a robust Monte Carlo Cross-Validation. As measurement of molecular weight is not precise even with standard lab methods, these represent acceptable accuracy. Optimal features selected for the prediction of molecular weight again highlighted the importance of stringent temperature control during the process. The better prediction of molecular weight for medical-grade PLA may be because the medical-grade PLA experiments were conducted in a much more controlled environment than those of the packaging-grade PLA, and also more extensive degradation was induced over the range of medical-grade trials. This work highlights feature selection algorithms as a powerful tool for process monitoring that should be more widely investigated for quality control in polymer processing industries. 

## Figures and Tables

**Table 1:** Detail of summarisation methods for packaging-grade PLA and medical-grade PLA.

Summary Methods to Predict Yield Stress		
Summary Methods	Summary Stats for Input Features	Size of the Data Set
Method 1 (six stats)	Mean, variance, kurtosis, skewness, max, and min value	n = 30, *p* = 3672
Method 2 (mean and var)	Mean and variance	n = 30, *p* = 1224
**Summary Methods to Predict Molecular Weight**		
Method 3 (mean—packaging grade)	Mean	n = 30, *p* = 612
Method 3 (mean—medical grade)	Mean	n = 62, *p* = 512

**Table 2:** Experiments for medical-grade PLA.

Temperature Profile (°C)				Feed Rate	Nozzle Type	Point Sample Taken for Mw Analysis	
Extruder Barrel Zone	Adapter	Die	Nozzle	g/h		Extruder exit	Spool
212	222	244	264	270, 300	A		yes
215	225	247	267	200, 220	B		yes
217	227	254	276	100, 150, 200, 250, 300, 350	C	yes	
218	228	249	269	320	C		yes
222	232	259	281	150, 200, 250, 300	C	yes	
223	233	254	276	100, 200, 300	D	yes	

**Table 3:** Comparison of RMSE values of all models for independent test set for prediction of yield stress for packaging-grade PLA (N_c = no. of components, N_s = no. of features selected).

Summary Methods	Method 1 (Six Summary Stats)	Method 2 (Mean and Variance)	Method 3 (Mean Only)
**Regression methods**			
Models	RMSE (MPa)	RMSE (MPa)	RMSE (MPa)
RF	3.02	2.979	2.148
PCR	5.533 (N_c = 5)	5.042(N_c = 2)	2.799 (N_c = 2)
PLS	6.532 (N_c = 4)	6.172 (N_c = 2)	2.683 (N_c = 2)
Ridge	6.906	9.386	8.180
**Feature selection methods**			
RFE-RF	1.07 (N_s = 10)	1.918 (N_s = 20)	1.773 (N_s = 11)
RFE-bagging	1.289 (N_s = 30)	1.73 (N_s = 29)	1.725 (N_s = 29)
FFS	1.407 (N_s = 14)	-	-
LASSO	2.25	9.990	9.79

**Table 4:** RMSE values of iPLS-RFE models for independent test set for summary method 1 for prediction of yield stress.

iPLS Followed by RFE (Method 1 with Six Summary Stats)		
Model	Features Selected	RMSE (MPa)
BiPLS-RFE-RF	43	1.156
BiPLS-RFE-bagging	18	0.911
FiPLS-RFE-RF	10	1.19
FiPLS-RFE-bagging	24	1.095

**Table 5:** Feature selection and regression methods to predict Mw for medical-grade and packaging-grade PLA (N_c = no. of components, N_s = no. of features selected).

Mean of Input Features										
Packaging-Grade PLA				Medical-Grade PLA						
	Result for Independent Test Set			Result for 80:20 Train Test Split			MC-CV with 200 Resampling			
Method	Features Selected	NRMSE	R^2^	Features Selected	NRMSE	R^2^	Features Selected	Mean NRMSE	SD of NRMSE	Mean R^2^
LASSO	N_s = 23	0.51	0.112	N_s = 41	0.102	0.926				
PCR	N_c = 3	0.428	0.021	N_c = 6	0.11	0.936	N_c = 3	0.193	0.120	0.433
PLS	N_c = 3	0.427	0.018	N_c = 6	0.114	0.93	N_c = 3	0.131	0.137	0.741
RFE-RF	N_s = 64	0.549	0.011	N_s = 9	0.134	0.863	N_s = 10	0.101	0.173	0.830
RFE-bagging	N_s = 90	0.496	0.045	N_s = 6	0.164	0.765				

## Data Availability

The experimental data and simulation models used in this research are available from the corresponding author upon request.
